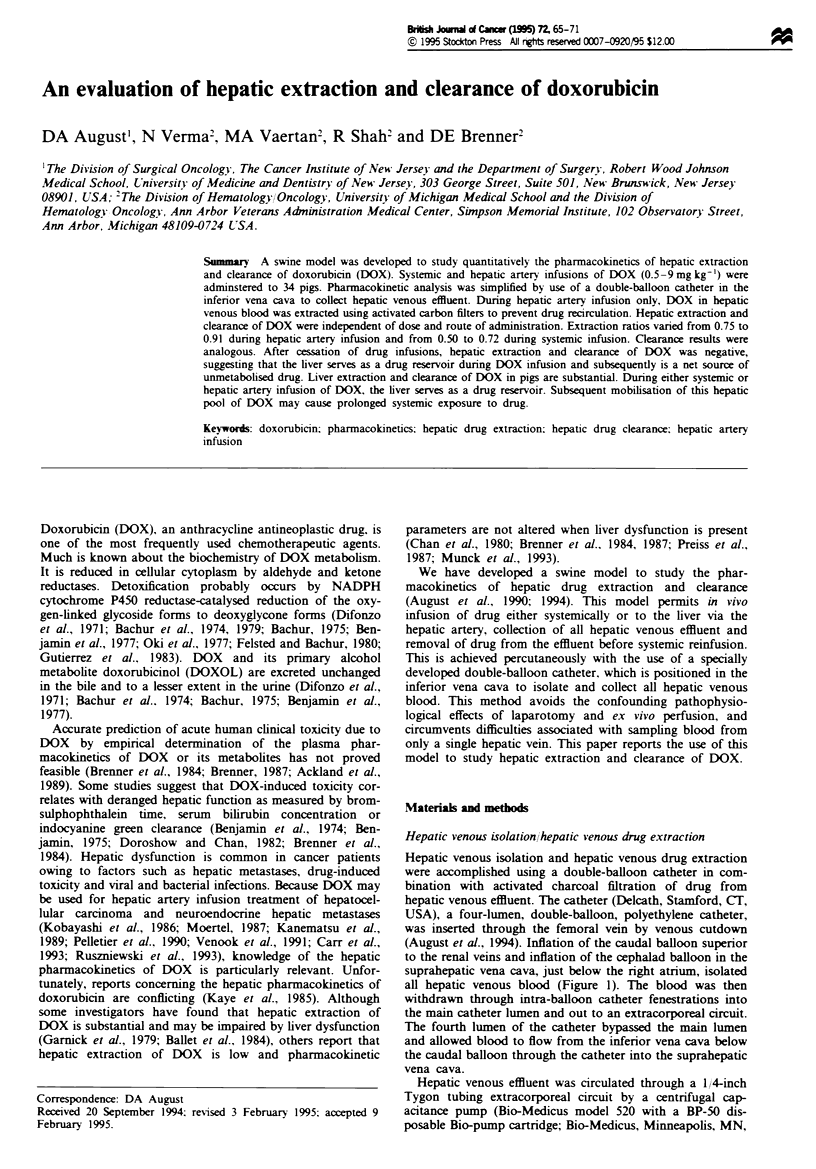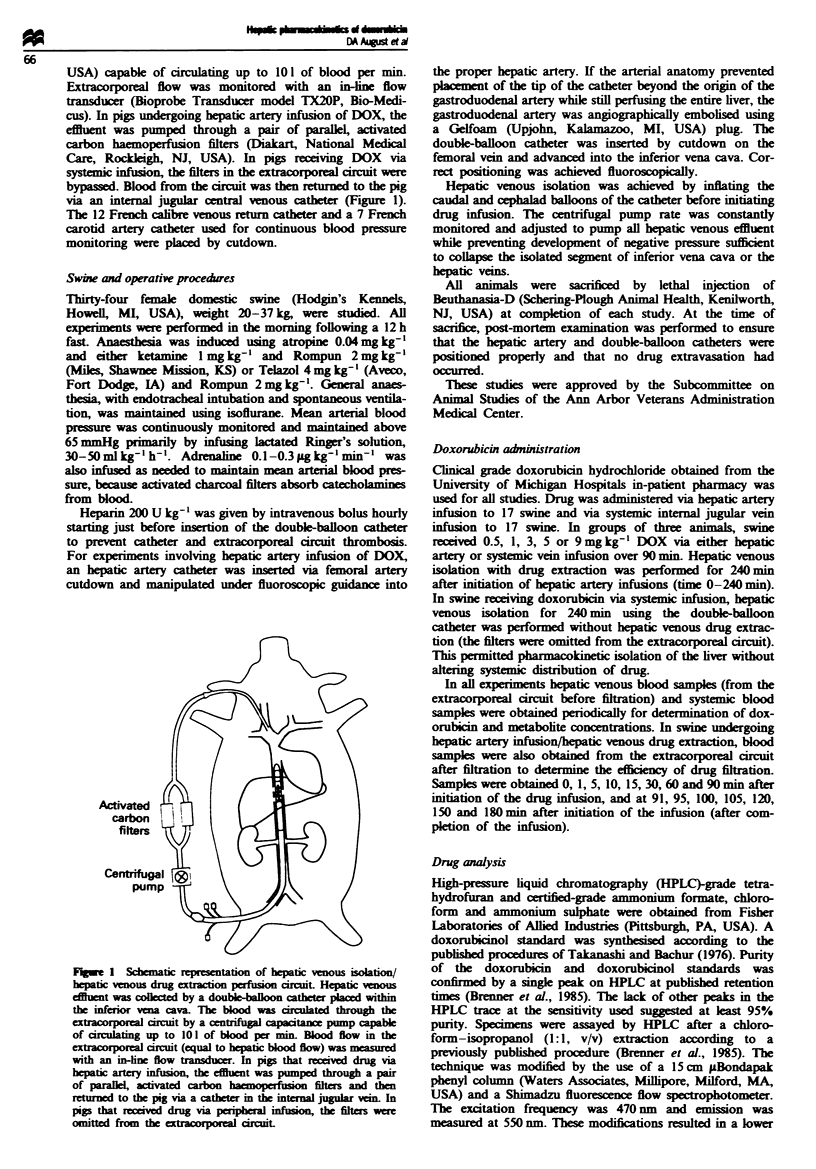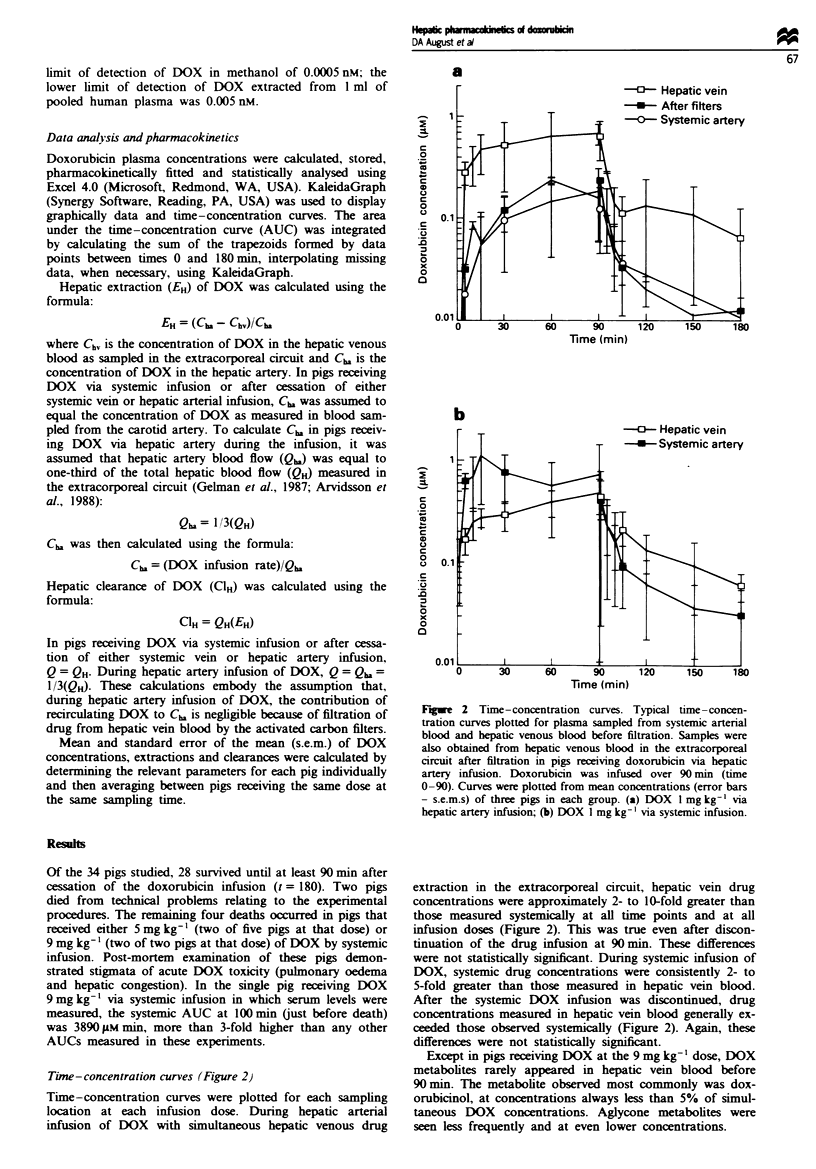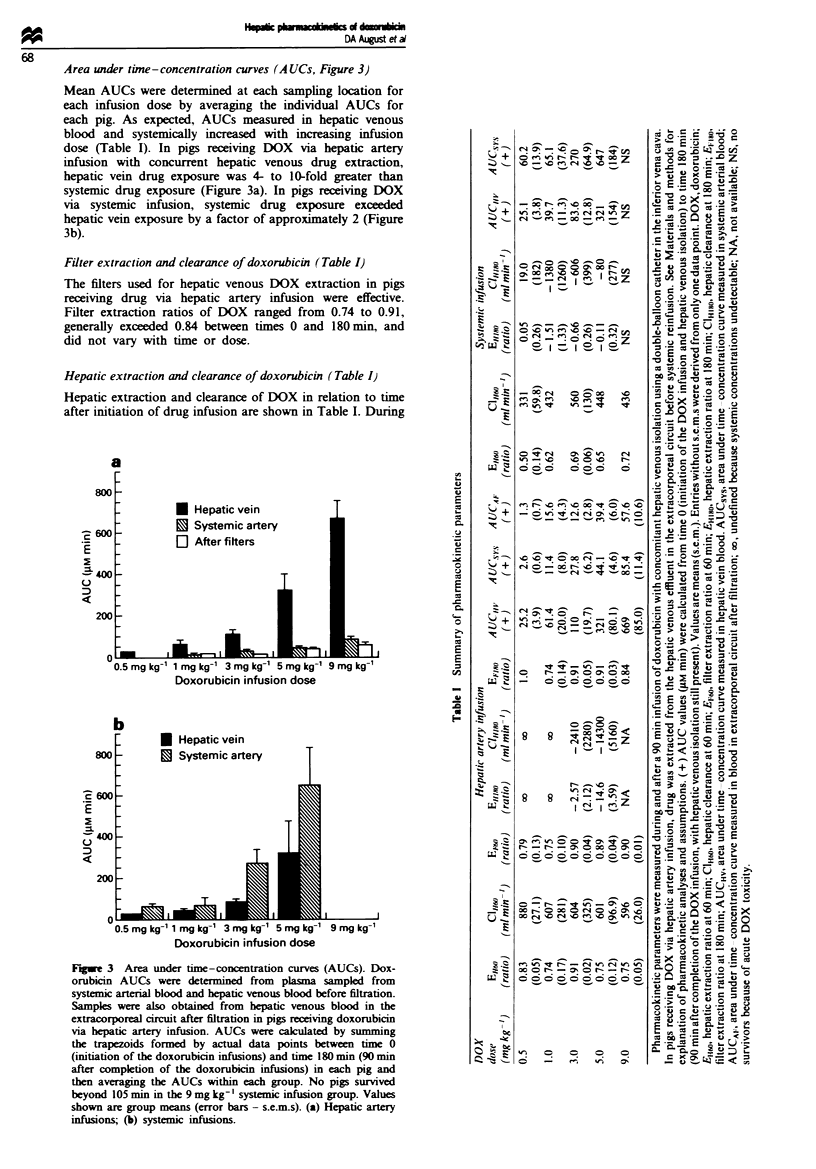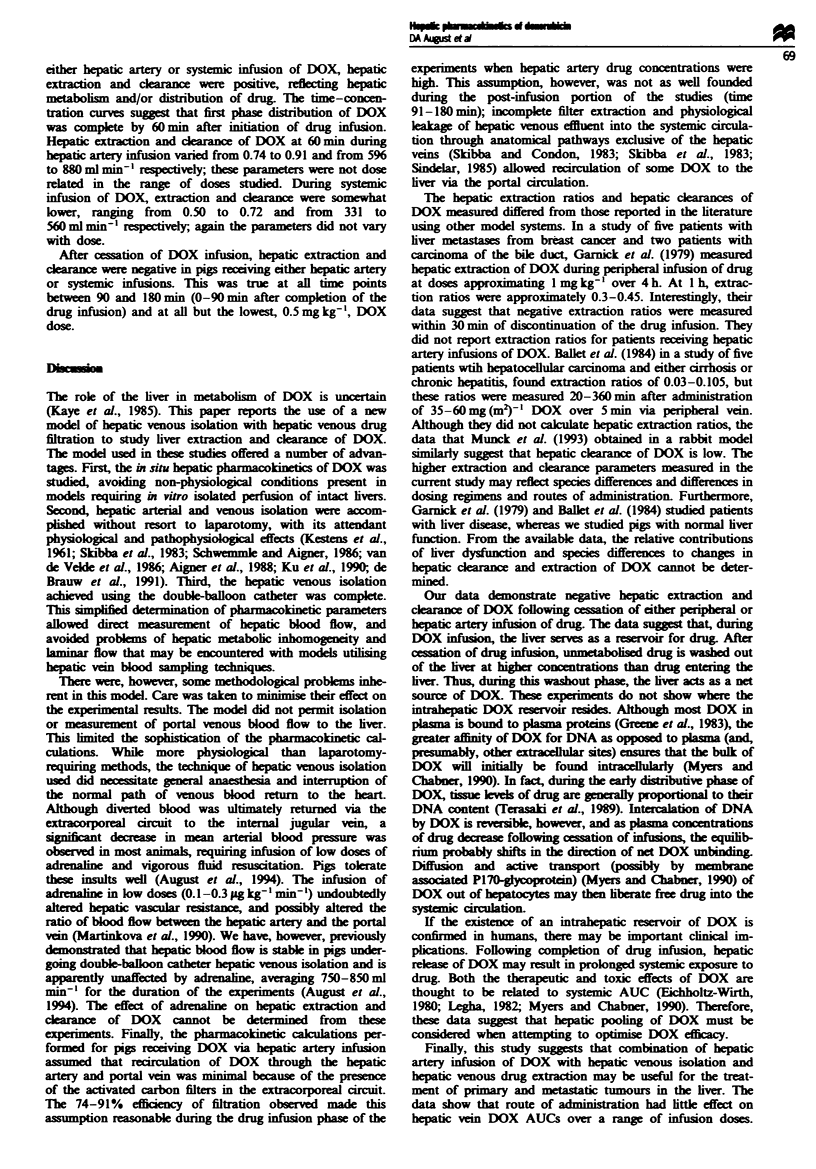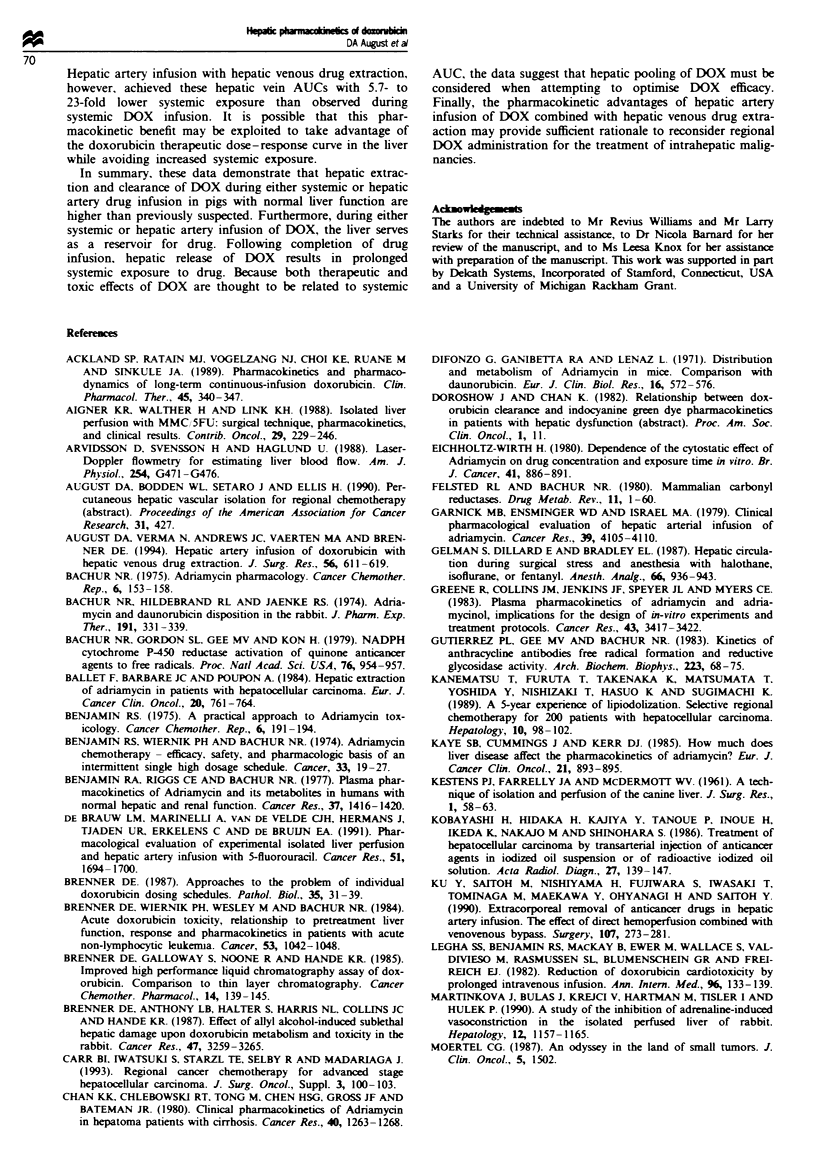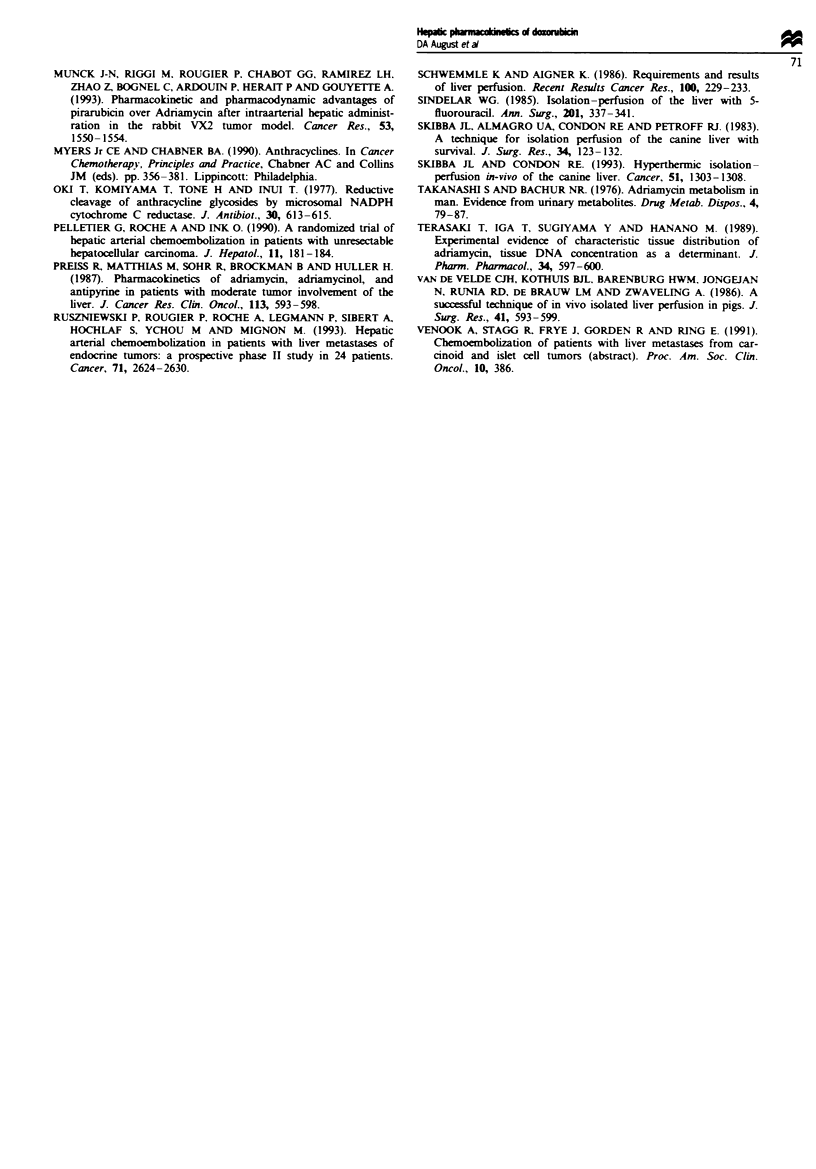# An evaluation of hepatic extraction and clearance of doxorubicin.

**DOI:** 10.1038/bjc.1995.278

**Published:** 1995-07

**Authors:** D. A. August, N. Verma, M. A. Vaertan, R. Shah, D. E. Brenner

**Affiliations:** Division of Surgical Oncology, Cancer Institute of New Jersey, University of Medicine and Dentistry of New Jersey 08901, USA.

## Abstract

A swine model was developed to study quantitatively the pharmacokinetics of hepatic extraction and clearance of doxorubicin (DOX). Systemic and hepatic artery infusions of DOX (0.5-9 mg kg-1) were administered to 34 pigs. Pharmacokinetic analysis was simplified by use of a double-balloon catheter in the inferior vena cava to collect hepatic venous effluent. During hepatic artery infusion only, DOX in hepatic venous blood was extracted using activated carbon filters to prevent drug recirculation. Hepatic extraction and clearance of DOX were independent of dose and route of administration. Extraction ratios varied from 0.75 to 0.91 during hepatic artery infusion and from 0.50 to 0.72 during systemic infusion. Clearance results were analogous. After cessation of drug infusions, hepatic extraction and clearance of DOX was negative, suggesting that the liver serves as a drug reservoir during DOX infusion and subsequently is a net source of unmetabolised drug. Liver extraction and clearance of DOX in pigs are substantial. During either systemic or hepatic artery infusion of DOX, the liver serves as a drug reservoir. Subsequent mobilisation of this hepatic pool of DOX may cause prolonged systemic exposure to drug.


© 1995 Stockton Press All rights reserved 0007–0920/95 $12.00

### An evaluation of hepatic extraction and clearance of doxorubicin

DA August[1], N Verma[2], MA Vaertan[2], R Shah[2] and DE Brenner[2]

[1]*The Division of Surgical Oncology, The Cancer Institute of New Jersey and the Department of Surgery, Robert Wood Johnson Medical School, University of Medicine and Dentistry of New Jersey, 303 George Street, Suite 501, New Brunswick, New Jersey 08901, USA;* [2]*The Division of Hematology/Oncology, University of Michigan Medical School and the Division of Hematology/Oncology, Ann Arbor Veterans Administration Medical Center, Simpson Memorial Institute, 102 Observatory Street, Ann Arbor, Michigan 48109-0724 USA.*

**Summary** A swine model was developed to study quantitatively the pharmacokinetics of hepatic extraction and clearance of doxorubicin (DOX). Systemic and hepatic artery infusions of DOX (0.5–9 mg $kg^{-1}$) were adminstered to 34 pigs. Pharmacokinetic analysis was simplified by use of a double-balloon catheter in the inferior vena cava to collect hepatic venous effluent. During hepatic artery infusion only, DOX in hepatic venous blood was extracted using activated carbon filters to prevent drug recirculation. Hepatic extraction and clearance of DOX were independent of dose and route of administration. Extraction ratios varied from 0.75 to 0.91 during hepatic artery infusion and from 0.50 to 0.72 during systemic infusion. Clearance results were analogous. After cessation of drug infusions, hepatic extraction and clearance of DOX was negative, suggesting that the liver serves as a drug reservoir during DOX infusion and subsequently is a net source of unmetabolised drug. Liver extraction and clearance of DOX in pigs are substantial. During either systemic or hepatic artery infusion of DOX, the liver serves as a drug reservoir. Subsequent mobilisation of this hepatic pool of DOX may cause prolonged systemic exposure to drug.

**Keywords**: doxorubicin; pharmacokinetics; hepatic drug extraction; hepatic drug clearance; hepatic artery infusion

Doxorubicin (DOX), an anthracycline antineoplastic drug, is one of the most frequently used chemotherapeutic agents. Much is known about the biochemistry of DOX metabolism. It is reduced in cellular cytoplasm by aldehyde and ketone reductases. Detoxification probably occurs by NADPH cytochrome P450 reductase-catalysed reduction of the oxygen-linked glycoside forms to deoxyglycone forms (Difonzo *et al.*, 1971; Bachur *et al.*, 1974, 1979; Bachur, 1975; Benjamin *et al.*, 1977; Oki *et al.*, 1977; Felsted and Bachur, 1980; Gutierrez *et al.*, 1983). DOX and its primary alcohol metabolite doxorubicinol (DOXOL) are excreted unchanged in the bile and to a lesser extent in the urine (Difonzo *et al.*, 1971; Bachur *et al.*, 1974; Bachur, 1975; Benjamin *et al.*, 1977).

Accurate prediction of acute human clinical toxicity due to DOX by empirical determination of the plasma pharmacokinetics of DOX or its metabolites has not proved feasible (Brenner *et al.*, 1984; Brenner, 1987; Ackland *et al.*, 1989). Some studies suggest that DOX-induced toxicity correlates with deranged hepatic function as measured by bromsulphophthalein time, serum bilirubin concentration or indocyanine green clearance (Benjamin *et al.*, 1974; Benjamin, 1975; Doroshow and Chan, 1982; Brenner *et al.*, 1984). Hepatic dysfunction is common in cancer patients owing to factors such as hepatic metastases, drug-induced toxicity and viral and bacterial infections. Because DOX may be used for hepatic artery infusion treatment of hepatocellular carcinoma and neuroendocrine hepatic metastases (Kobayashi *et al.*, 1986; Moertel, 1987; Kanematsu *et al.*, 1989; Pelletier *et al.*, 1990; Venook *et al.*, 1991; Carr *et al.*, 1993; Ruszniewski *et al.*, 1993), knowledge of the hepatic pharmacokinetics of DOX is particularly relevant. Unfortunately, reports concerning the hepatic pharmacokinetics of doxorubicin are conflicting (Kaye *et al.*, 1985). Although some investigators have found that hepatic extraction of DOX is substantial and may be impaired by liver dysfunction (Garnick *et al.*, 1979; Ballet *et al.*, 1984), others report that hepatic extraction of DOX is low and pharmacokinetic parameters are not altered when liver dysfunction is present (Chan *et al.*, 1980; Brenner *et al.*, 1984, 1987; Preiss *et al.*, 1987; Munck *et al.*, 1993).

We have developed a swine model to study the pharmacokinetics of hepatic drug extraction and clearance (August *et al.*, 1990; 1994). This model permits *in vivo* infusion of drug either systemically or to the liver via the hepatic artery, collection of all hepatic venous effluent and removal of drug from the effluent before systemic reinfusion. This is achieved percutaneously with the use of a specially developed double-balloon catheter, which is positioned in the inferior vena cava to isolate and collect all hepatic venous blood. This method avoids the confounding pathophysiological effects of laparotomy and *ex vivo* perfusion, and circumvents difficulties associated with sampling blood from only a single hepatic vein. This paper reports the use of this model to study hepatic extraction and clearance of DOX.

### Materials and methods

### *Hepatic venous isolation/hepatic venous drug extraction*

Hepatic venous isolation and hepatic venous drug extraction were accomplished using a double-balloon catheter in combination with activated charcoal filtration of drug from hepatic venous effluent. The catheter (Delcath, Stamford, CT, USA), a four-lumen, double-balloon, polyethylene catheter, was inserted through the femoral vein by venous cutdown (August *et al.*, 1994). Inflation of the caudal balloon superior to the renal veins and inflation of the cephalad balloon in the suprahepatic vena cava, just below the right atrium, isolated all hepatic venous blood (Figure 1). The blood was then withdrawn through intra-balloon catheter fenestrations into the main catheter lumen and out to an extracorporeal circuit. The fourth lumen of the catheter bypassed the main lumen and allowed blood to flow from the inferior vena cava below the caudal balloon through the catheter into the suprahepatic vena cava.

Hepatic venous effluent was circulated through a 1/4-inch Tygon tubing extracorporeal circuit by a centrifugal capacitance pump (Bio-Medicus model 520 with a BP-50 disposable Bio-pump cartridge; Bio-Medicus, Minneapolis, MN,

Correspondence: DA August
Received 20 September 1994; revised 3 February 1995; accepted 9 February 1995.

USA) capable of circulating up to 10 l of blood per min. Extracorporeal flow was monitored with an in-line flow transducer (Bioprobe Transducer model TX20P, Bio-Medicus). In pigs undergoing hepatic artery infusion of DOX, the effluent was pumped through a pair of parallel, activated carbon haemoperfusion filters (Diakart, National Medical Care, Rockleigh, NJ, USA). In pigs receiving DOX via systemic infusion, the filters in the extracorporeal circuit were bypassed. Blood from the circuit was then returned to the pig via an internal jugular central venous catheter (Figure 1). The 12 French calibre venous return catheter and a 7 French carotid artery catheter used for continuous blood pressure monitoring were placed by cutdown.

*Swine and operative procedures*

Thirty-four female domestic swine (Hodgin's Kennels, Howell, MI, USA), weight 20–37 kg, were studied. All experiments were performed in the morning following a 12 h fast. Anaesthesia was induced using atropine 0.04 mg $kg^{-1}$ and either ketamine 1 mg $kg^{-1}$ and Rompun 2 mg $kg^{-1}$ (Miles, Shawnee Mission, KS) or Telazol 4 mg $kg^{-1}$ (Aveco, Fort Dodge, IA) and Rompun 2 mg $kg^{-1}$. General anaesthesia, with endotracheal intubation and spontaneous ventilation, was maintained using isoflurane. Mean arterial blood pressure was continuously monitored and maintained above 65 mmHg primarily by infusing lactated Ringer's solution, 30–50 ml $kg^{-1}$ $h^{-1}$. Adrenaline 0.1–0.3 $\mu$g $kg^{-1}$ $min^{-1}$ was also infused as needed to maintain mean arterial blood pressure, because activated charcoal filters absorb catecholamines from blood.

Heparin 200 U $kg^{-1}$ was given by intravenous bolus hourly starting just before insertion of the double-balloon catheter to prevent catheter and extracorporeal circuit thrombosis. For experiments involving hepatic artery infusion of DOX, an hepatic artery catheter was inserted via femoral artery cutdown and manipulated under fluoroscopic guidance into

**Figure 1** Schematic representation of hepatic venous isolation/hepatic venous drug extraction perfusion circuit. Hepatic venous effluent was collected by a double-balloon catheter placed within the inferior vena cava. The blood was circulated through the extracorporeal circuit by a centrifugal capacitance pump capable of circulating up to 10 l of blood per min. Blood flow in the extracorporeal circuit (equal to hepatic blood flow) was measured with an in-line flow transducer. In pigs that received drug via hepatic artery infusion, the effluent was pumped through a pair of parallel, activated carbon haemoperfusion filters and then returned to the pig via a catheter in the internal jugular vein. In pigs that received drug via peripheral infusion, the filters were omitted from the extracorporeal circuit.

the proper hepatic artery. If the arterial anatomy prevented placement of the tip of the catheter beyond the origin of the gastroduodenal artery while still perfusing the entire liver, the gastroduodenal artery was angiographically embolised using a Gelfoam (Upjohn, Kalamazoo, MI, USA) plug. The double-balloon catheter was inserted by cutdown on the femoral vein and advanced into the inferior vena cava. Correct positioning was achieved fluoroscopically.

Hepatic venous isolation was achieved by inflating the caudal and cephalad balloons of the catheter before initiating drug infusion. The centrifugal pump rate was constantly monitored and adjusted to pump all hepatic venous effluent while preventing development of negative pressure sufficient to collapse the isolated segment of inferior vena cava or the hepatic veins.

All animals were sacrificed by lethal injection of Beuthanasia-D (Schering-Plough Animal Health, Kenilworth, NJ, USA) at completion of each study. At the time of sacrifice, post-mortem examination was performed to ensure that the hepatic artery and double-balloon catheters were positioned properly and that no drug extravasation had occurred.

These studies were approved by the Subcommittee on Animal Studies of the Ann Arbor Veterans Administration Medical Center.

*Doxorubicin administration*

Clinical grade doxorubicin hydrochloride obtained from the University of Michigan Hospitals in-patient pharmacy was used for all studies. Drug was administered via hepatic artery infusion to 17 swine and via systemic internal jugular vein infusion to 17 swine. In groups of three animals, swine received 0.5, 1, 3, 5 or 9 mg $kg^{-1}$ DOX via either hepatic artery or systemic vein infusion over 90 min. Hepatic venous isolation with drug extraction was performed for 240 min after initiation of hepatic artery infusions (time 0–240 min). In swine receiving doxorubicin via systemic infusion, hepatic venous isolation for 240 min using the double-balloon catheter was performed without hepatic venous drug extraction (the filters were omitted from the extracorporeal circuit). This permitted pharmacokinetic isolation of the liver without altering systemic distribution of drug.

In all experiments hepatic venous blood samples (from the extracorporeal circuit before filtration) and systemic blood samples were obtained periodically for determination of doxorubicin and metabolite concentrations. In swine undergoing hepatic artery infusion/hepatic venous drug extraction, blood samples were also obtained from the extracorporeal circuit after filtration to determine the efficiency of drug filtration. Samples were obtained 0, 1, 5, 10, 15, 30, 60 and 90 min after initiation of the drug infusion, and at 91, 95, 100, 105, 120, 150 and 180 min after initiation of the infusion (after completion of the infusion).

*Drug analysis*

High-pressure liquid chromatography (HPLC)-grade tetrahydrofuran and certified-grade ammonium formate, chloroform and ammonium sulphate were obtained from Fisher Laboratories of Allied Industries (Pittsburgh, PA, USA). A doxorubicinol standard was synthesised according to the published procedures of Takanashi and Bachur (1976). Purity of the doxorubicin and doxorubicinol standards was confirmed by a single peak on HPLC at published retention times (Brenner et al., 1985). The lack of other peaks in the HPLC trace at the sensitivity used suggested at least 95% purity. Specimens were assayed by HPLC after a chloroform–isopropanol (1:1, v/v) extraction according to a previously published procedure (Brenner et al., 1985). The technique was modified by the use of a 15 cm $\mu$Bondapak phenyl column (Waters Associates, Millipore, Milford, MA, USA) and a Shimadzu fluorescence flow spectrophotometer. The excitation frequency was 470 nm and emission was measured at 550 nm. These modifications resulted in a lower limit of detection of DOX in methanol of 0.0005 nM; the lower limit of detection of DOX extracted from 1 ml of pooled human plasma was 0.005 nM.

*Data analysis and pharmacokinetics*

Doxorubicin plasma concentrations were calculated, stored, pharmacokinetically fitted and statistically analysed using Excel 4.0 (Microsoft, Redmond, WA, USA). KaleidaGraph (Synergy Software, Reading, PA, USA) was used to display graphically data and time–concentration curves. The area under the time–concentration curve (AUC) was integrated by calculating the sum of the trapezoids formed by data points between times 0 and 180 min, interpolating missing data, when necessary, using KaleidaGraph.

Hepatic extraction ($E_H$) of DOX was calculated using the formula:

$$E_H = (C_{ha} - C_{hv})/C_{ha}$$

where $C_{hv}$ is the concentration of DOX in the hepatic venous blood as sampled in the extracorporeal circuit and $C_{ha}$ is the concentration of DOX in the hepatic artery. In pigs receiving DOX via systemic infusion or after cessation of either systemic vein or hepatic arterial infusion, $C_{ha}$ was assumed to equal the concentration of DOX as measured in blood sampled from the carotid artery. To calculate $C_{ha}$ in pigs receiving DOX via hepatic artery during the infusion, it was assumed that hepatic artery blood flow ($Q_{ha}$) was equal to one-third of the total hepatic blood flow ($Q_H$) measured in the extracorporeal circuit (Gelman *et al.*, 1987; Arvidsson *et al.*, 1988):

$$Q_{ha} = 1/3(Q_H)$$

$C_{ha}$ was then calculated using the formula:

$$C_{ha} = (\text{DOX infusion rate})/Q_{ha}$$

Hepatic clearance of DOX ($Cl_H$) was calculated using the formula:

$$Cl_H = Q_H(E_H)$$

In pigs receiving DOX via systemic infusion or after cessation of either systemic vein or hepatic artery infusion, $Q = Q_H$. During hepatic artery infusion of DOX, $Q = Q_{ha} = 1/3(Q_H)$. These calculations embody the assumption that, during hepatic artery infusion of DOX, the contribution of recirculating DOX to $C_{ha}$ is negligible because of filtration of drug from hepatic vein blood by the activated carbon filters.

Mean and standard error of the mean (s.e.m.) of DOX concentrations, extractions and clearances were calculated by determining the relevant parameters for each pig individually and then averaging between pigs receiving the same dose at the same sampling time.

**Figure 2** Time–concentration curves. Typical time–concentration curves plotted for plasma sampled from systemic arterial blood and hepatic venous blood before filtration. Samples were also obtained from hepatic venous blood in the extracorporeal circuit after filtration in pigs receiving doxorubicin via hepatic artery infusion. Doxorubicin was infused over 90 min (time 0–90). Curves were plotted from mean concentrations (error bars – s.e.m.s) of three pigs in each group. (**a**) DOX 1 mg $kg^{-1}$ via hepatic artery infusion; (**b**) DOX 1 mg $kg^{-1}$ via systemic infusion.

### Results

Of the 34 pigs studied, 28 survived until at least 90 min after cessation of the doxorubicin infusion ($t = 180$). Two pigs died from technical problems relating to the experimental procedures. The remaining four deaths occurred in pigs that received either 5 mg $kg^{-1}$ (two of five pigs at that dose) or 9 mg $kg^{-1}$ (two of two pigs at that dose) of DOX by systemic infusion. Post-mortem examination of these pigs demonstrated stigmata of acute DOX toxicity (pulmonary oedema and hepatic congestion). In the single pig receiving DOX 9 mg $kg^{-1}$ via systemic infusion in which serum levels were measured, the systemic AUC at 100 min (just before death) was 3890 μM min, more than 3-fold higher than any other AUCs measured in these experiments.

*Time–concentration curves (Figure 2)*

Time–concentration curves were plotted for each sampling location at each infusion dose. During hepatic arterial infusion of DOX with simultaneous hepatic venous drug extraction in the extracorporeal circuit, hepatic vein drug concentrations were approximately 2- to 10-fold greater than those measured systemically at all time points and at all infusion doses (Figure 2). This was true even after discontinuation of the drug infusion at 90 min. These differences were not statistically significant. During systemic infusion of DOX, systemic drug concentrations were consistently 2- to 5-fold greater than those measured in hepatic vein blood. After the systemic DOX infusion was discontinued, drug concentrations measured in hepatic vein blood generally exceeded those observed systemically (Figure 2). Again, these differences were not statistically significant.

Except in pigs receiving DOX at the 9 mg $kg^{-1}$ dose, DOX metabolites rarely appeared in hepatic vein blood before 90 min. The metabolite observed most commonly was doxorubicinol, at concentrations always less than 5% of simultaneous DOX concentrations. Aglycone metabolites were seen less frequently and at even lower concentrations.

*Area under time–concentration curves (AUCs, Figure 3)*

Mean AUCs were determined at each sampling location for each infusion dose by averaging the individual AUCs for each pig. As expected, AUCs measured in hepatic venous blood and systemically increased with increasing infusion dose (Table I). In pigs receiving DOX via hepatic artery infusion with concurrent hepatic venous drug extraction, hepatic vein drug exposure was 4- to 10-fold greater than systemic drug exposure (Figure 3a). In pigs receiving DOX via systemic infusion, systemic drug exposure exceeded hepatic vein exposure by a factor of approximately 2 (Figure 3b).

*Filter extraction and clearance of doxorubicin (Table I)*

The filters used for hepatic venous DOX extraction in pigs receiving drug via hepatic artery infusion were effective. Filter extraction ratios of DOX ranged from 0.74 to 0.91, generally exceeded 0.84 between times 0 and 180 min, and did not vary with time or dose.

*Hepatic extraction and clearance of doxorubicin (Table I)*

Hepatic extraction and clearance of DOX in relation to time after initiation of drug infusion are shown in Table I. During

**Figure 3** Area under time–concentration curves (AUCs). Doxorubicin AUCs were determined from plasma sampled from systemic arterial blood and hepatic venous blood before filtration. Samples were also obtained from hepatic venous blood in the extracorporeal circuit after filtration in pigs receiving doxorubicin via hepatic artery infusion. AUCs were calculated by summing the trapezoids formed by actual data points between time 0 (initiation of the doxorubicin infusions) and time 180 min (90 min after completion of the doxorubicin infusions) in each pig and then averaging the AUCs within each group. No pigs survived beyond 105 min in the 9 mg kg$^{-1}$ systemic infusion group. Values shown are group means (error bars – s.e.m.s). (**a**) Hepatic artery infusions; (**b**) systemic infusions.

either hepatic artery or systemic infusion of DOX, hepatic extraction and clearance were positive, reflecting hepatic metabolism and/or distribution of drug. The time–concentration curves suggest that first phase distribution of DOX was complete by 60 min after initiation of drug infusion. Hepatic extraction and clearance of DOX at 60 min during hepatic artery infusion varied from 0.74 to 0.91 and from 596 to 880 ml $min^{-1}$ respectively; these parameters were not dose related in the range of doses studied. During systemic infusion of DOX, extraction and clearance were somewhat lower, ranging from 0.50 to 0.72 and from 331 to 560 ml $min^{-1}$ respectively; again the parameters did not vary with dose.

After cessation of DOX infusion, hepatic extraction and clearance were negative in pigs receiving either hepatic artery or systemic infusions. This was true at all time points between 90 and 180 min (0–90 min after completion of the drug infusion) and at all but the lowest, 0.5 mg $kg^{-1}$, DOX dose.

### Discussion

The role of the liver in metabolism of DOX is uncertain (Kaye *et al.*, 1985). This paper reports the use of a new model of hepatic venous isolation with hepatic venous drug filtration to study liver extraction and clearance of DOX. The model used in these studies offered a number of advantages. First, the *in situ* hepatic pharmacokinetics of DOX was studied, avoiding non-physiological conditions present in models requiring *in vitro* isolated perfusion of intact livers. Second, hepatic arterial and venous isolation were accomplished without resort to laparotomy, with its attendant physiological and pathophysiological effects (Kestens *et al.*, 1961; Skibba *et al.*, 1983; Schwemmle and Aigner, 1986; van de Velde *et al.*, 1986; Aigner *et al.*, 1988; Ku *et al.*, 1990; de Brauw *et al.*, 1991). Third, the hepatic venous isolation achieved using the double-balloon catheter was complete. This simplified determination of pharmacokinetic parameters allowed direct measurement of hepatic blood flow, and avoided problems of hepatic metabolic inhomogeneity and laminar flow that may be encountered with models utilising hepatic vein blood sampling techniques.

There were, however, some methodological problems inherent in this model. Care was taken to minimise their effect on the experimental results. The model did not permit isolation or measurement of portal venous blood flow to the liver. This limited the sophistication of the pharmacokinetic calculations. While more physiological than laparotomy-requiring methods, the technique of hepatic venous isolation used did necessitate general anaesthesia and interruption of the normal path of venous blood return to the heart. Although diverted blood was ultimately returned via the extracorporeal circuit to the internal jugular vein, a significant decrease in mean arterial blood pressure was observed in most animals, requiring infusion of low doses of adrenaline and vigorous fluid resuscitation. Pigs tolerate these insults well (August *et al.*, 1994). The infusion of adrenaline in low doses (0.1–0.3 $\mu g\ kg^{-1}\ min^{-1}$) undoubtedly altered hepatic vascular resistance, and possibly altered the ratio of blood flow between the hepatic artery and the portal vein (Martinkova *et al.*, 1990). We have, however, previously demonstrated that hepatic blood flow is stable in pigs undergoing double-balloon catheter hepatic venous isolation and is apparently unaffected by adrenaline, averaging 750–850 ml $min^{-1}$ for the duration of the experiments (August *et al.*, 1994). The effect of adrenaline on hepatic extraction and clearance of DOX cannot be determined from these experiments. Finally, the pharmacokinetic calculations performed for pigs receiving DOX via hepatic artery infusion assumed that recirculation of DOX through the hepatic artery and portal vein was minimal because of the presence of the activated carbon filters in the extracorporeal circuit. The 74–91% efficiency of filtration observed made this assumption reasonable during the drug infusion phase of the experiments when hepatic artery drug concentrations were high. This assumption, however, was not as well founded during the post-infusion portion of the studies (time 91–180 min); incomplete filter extraction and physiological leakage of hepatic venous effluent into the systemic circulation through anatomical pathways exclusive of the hepatic veins (Skibba and Condon, 1983; Skibba *et al.*, 1983; Sindelar, 1985) allowed recirculation of some DOX to the liver via the portal circulation.

The hepatic extraction ratios and hepatic clearances of DOX measured differed from those reported in the literature using other model systems. In a study of five patients with liver metastases from breast cancer and two patients with carcinoma of the bile duct, Garnick *et al.* (1979) measured hepatic extraction of DOX during peripheral infusion of drug at doses approximating 1 mg $kg^{-1}$ over 4 h. At 1 h, extraction ratios were approximately 0.3–0.45. Interestingly, their data suggest that negative extraction ratios were measured within 30 min of discontinuation of the drug infusion. They did not report extraction ratios for patients receiving hepatic artery infusions of DOX. Ballet *et al.* (1984) in a study of five patients wtih hepatocellular carcinoma and either cirrhosis or chronic hepatitis, found extraction ratios of 0.03–0.105, but these ratios were measured 20–360 min after administration of 35–60 mg $(m^2)^{-1}$ DOX over 5 min via peripheral vein. Although they did not calculate hepatic extraction ratios, the data that Munck *et al.* (1993) obtained in a rabbit model similarly suggest that hepatic clearance of DOX is low. The higher extraction and clearance parameters measured in the current study may reflect species differences and differences in dosing regimens and routes of administration. Furthermore, Garnick *et al.* (1979) and Ballet *et al.* (1984) studied patients with liver disease, whereas we studied pigs with normal liver function. From the available data, the relative contributions of liver dysfunction and species differences to changes in hepatic clearance and extraction of DOX cannot be determined.

Our data demonstrate negative hepatic extraction and clearance of DOX following cessation of either peripheral or hepatic artery infusion of drug. The data suggest that, during DOX infusion, the liver serves as a reservoir for drug. After cessation of drug infusion, unmetabolised drug is washed out of the liver at higher concentrations than drug entering the liver. Thus, during this washout phase, the liver acts as a net source of DOX. These experiments do not show where the intrahepatic DOX reservoir resides. Although most DOX in plasma is bound to plasma proteins (Greene *et al.*, 1983), the greater affinity of DOX for DNA as opposed to plasma (and, presumably, other extracellular sites) ensures that the bulk of DOX will initially be found intracellularly (Myers and Chabner, 1990). In fact, during the early distributive phase of DOX, tissue levels of drug are generally proportional to their DNA content (Terasaki *et al.*, 1989). Intercalation of DNA by DOX is reversible, however, and as plasma concentrations of drug decrease following cessation of infusions, the equilibrium probably shifts in the direction of net DOX unbinding. Diffusion and active transport (possibly by membrane associated P170-glycoprotein) (Myers and Chabner, 1990) of DOX out of hepatocytes may then liberate free drug into the systemic circulation.

If the existence of an intrahepatic reservoir of DOX is confirmed in humans, there may be important clinical implications. Following completion of drug infusion, hepatic release of DOX may result in prolonged systemic exposure to drug. Both the therapeutic and toxic effects of DOX are thought to be related to systemic AUC (Eichholtz-Wirth, 1980; Legha, 1982; Myers and Chabner, 1990). Therefore, these data suggest that hepatic pooling of DOX must be considered when attempting to optimise DOX efficacy.

Finally, this study suggests that combination of hepatic artery infusion of DOX with hepatic venous isolation and hepatic venous drug extraction may be useful for the treatment of primary and metastatic tumours in the liver. The data show that route of administration had little effect on hepatic vein DOX AUCs over a range of infusion doses.

Hepatic artery infusion with hepatic venous drug extraction, however, achieved these hepatic vein AUCs with 5.7- to 23-fold lower systemic exposure than observed during systemic DOX infusion. It is possible that this pharmacokinetic benefit may be exploited to take advantage of the doxorubicin therapeutic dose-response curve in the liver while avoiding increased systemic exposure.

In summary, these data demonstrate that hepatic extraction and clearance of DOX during either systemic or hepatic artery drug infusion in pigs with normal liver function are higher than previously suspected. Furthermore, during either systemic or hepatic artery infusion of DOX, the liver serves as a reservoir for drug. Following completion of drug infusion, hepatic release of DOX results in prolonged systemic exposure to drug. Because both therapeutic and toxic effects of DOX are thought to be related to systemic AUC, the data suggest that hepatic pooling of DOX must be considered when attempting to optimise DOX efficacy. Finally, the pharmacokinetic advantages of hepatic artery infusion of DOX combined with hepatic venous drug extraaction may provide sufficient rationale to reconsider regional DOX administration for the treatment of intrahepatic malignancies.

**Acknowledgements**

The authors are indebted to Mr Revius Williams and Mr Larry Starks for their technical assistance, to Dr Nicola Barnard for her review of the manuscript, and to Ms Leesa Knox for her assistance with preparation of the manuscript. This work was supported in part by Delcath Systems, Incorporated of Stamford, Connecticut, USA and a University of Michigan Rackham Grant.

**References**

ACKLAND SP, RATAIN MJ, VOGELZANG NJ, CHOI KE, RUANE M AND SINKULE JA. (1989). Pharmacokinetics and pharmacodynamics of long-term continuous-infusion doxorubicin. *Clin. Pharmacol. Ther.*, **45**, 340-347.

AIGNER KR, WALTHER H AND LINK KH. (1988). Isolated liver perfusion with MMC/5FU: surgical technique, pharmacokinetics, and clinical results. *Contrib. Oncol.*, **29**, 229-246.

ARVIDSSON D, SVENSSON H AND HAGLUND U. (1988). Laser-Doppler flowmetry for estimating liver blood flow. *Am. J. Physiol.*, **254**, G471-G476.

AUGUST DA, BODDEN WL, SETARO J AND ELLIS H. (1990). Percutaneous hepatic vascular isolation for regional chemotherapy (abstract). *Proceedings of the American Association for Cancer Research*, **31**, 427.

AUGUST DA, VERMA N, ANDREWS JC, VAERTEN MA AND BRENNER DE. (1994). Hepatic artery infusion of doxorubicin with hepatic venous drug extraction. *J. Surg. Res.*, **56**, 611-619.

BACHUR NR. (1975). Adriamycin pharmacology. *Cancer Chemother. Rep.*, **6**, 153-158.

BACHUR NR, HILDEBRAND RL AND JAENKE RS. (1974). Adriamycin and daunorubicin disposition in the rabbit. *J. Pharm. Exp. Ther.*, **191**, 331-339.

BACHUR NR, GORDON SL, GEE MV AND KON H. (1979). NADPH cytochrome P-450 reductase activation of quinone anticancer agents to free radicals. *Proc. Natl Acad. Sci. USA*, **76**, 954-957.

BALLET F, BARBARE JC AND POUPON A. (1984). Hepatic extraction of adriamycin in patients with hepatocellular carcinoma. *Eur. J. Cancer Clin. Oncol.*, **20**, 761-764.

BENJAMIN RS. (1975). A practical approach to Adriamycin toxicology. *Cancer Chemother. Rep.*, **6**, 191-194.

BENJAMIN RS, WIERNIK PH AND BACHUR NR. (1974). Adriamycin chemotherapy - efficacy, safety, and pharmacologic basis of an intermittent single high dosage schedule. *Cancer*, **33**, 19-27.

BENJAMIN RA, RIGGS CE AND BACHUR NR. (1977). Plasma pharmacokinetics of Adriamycin and its metabolites in humans with normal hepatic and renal function. *Cancer Res.*, **37**, 1416-1420.

DE BRAUW LM, MARINELLI A, VAN DE VELDE CJH, HERMANS J, TJADEN UR, ERKELENS C AND DE BRUIJN EA. (1991). Pharmacological evaluation of experimental isolated liver perfusion and hepatic artery infusion with 5-fluorouracil. *Cancer Res.*, **51**, 1694-1700.

BRENNER DE. (1987). Approaches to the problem of individual doxorubicin dosing schedules. *Pathol. Biol.*, **35**, 31-39.

BRENNER DE, WIERNIK PH, WESLEY M AND BACHUR NR. (1984). Acute doxorubicin toxicity, relationship to pretreatment liver function, response and pharmacokinetics in patients with acute non-lymphocytic leukemia. *Cancer*, **53**, 1042-1048.

BRENNER DE, GALLOWAY S, NOONE R AND HANDE KR. (1985). Improved high performance liquid chromatography assay of doxorubicin. Comparison to thin layer chromatography. *Cancer Chemother. Pharmacol.*, **14**, 139-145.

BRENNER DE, ANTHONY LB, HALTER S, HARRIS NL, COLLINS JC AND HANDE KR. (1987). Effect of allyl alcohol-induced sublethal hepatic damage upon doxorubicin metabolism and toxicity in the rabbit. *Cancer Res.*, **47**, 3259-3265.

CARR BI, IWATSUKI S, STARZL TE, SELBY R AND MADARIAGA J. (1993). Regional cancer chemotherapy for advanced stage hepatocellular carcinoma. *J. Surg. Oncol.*, Suppl. **3**, 100-103.

CHAN KK, CHLEBOWSKI RT, TONG M, CHEN HSG, GROSS JF AND BATEMAN JR. (1980). Clinical pharmacokinetics of Adriamycin in hepatoma patients with cirrhosis. *Cancer Res.*, **40**, 1263-1268.

DIFONZO G, GANIBETTA RA AND LENAZ L. (1971). Distribution and metabolism of Adriamycin in mice. Comparison with daunorubicin. *Eur. J. Clin. Biol. Res.*, **16**, 572-576.

DOROSHOW J AND CHAN K. (1982). Relationship between doxorubicin clearance and indocyanine green dye pharmacokinetics in patients with hepatic dysfunction (abstract). *Proc. Am. Soc. Clin. Oncol.*, **1**, 11.

EICHHOLTZ-WIRTH H. (1980). Dependence of the cytostatic effect of Adriamycin on drug concentration and exposure time *in vitro*. *Br. J. Cancer*, **41**, 886-891.

FELSTED RL AND BACHUR NR. (1980). Mammalian carbonyl reductases. *Drug Metab. Rev.*, **11**, 1-60.

GARNICK MB, ENSMINGER WD AND ISRAEL MA. (1979). Clinical pharmacological evaluation of hepatic arterial infusion of adriamycin. *Cancer Res.*, **39**, 4105-4110.

GELMAN S, DILLARD E AND BRADLEY EL. (1987). Hepatic circulation during surgical stress and anesthesia with halothane, isoflurane, or fentanyl. *Anesth. Analg.*, **66**, 936-943.

GREENE R, COLLINS JM, JENKINS JF, SPEYER JL AND MYERS CE. (1983). Plasma pharmacokinetics of adriamycin and adriamycinol, implications for the design of *in-vitro* experiments and treatment protocols. *Cancer Res.*, **43**, 3417-3422.

GUTIERREZ PL, GEE MV AND BACHUR NR. (1983). Kinetics of anthracycline antibodies free radical formation and reductive glycosidase activity. *Arch. Biochem. Biophys.*, **223**, 68-75.

KANEMATSU T, FURUTA T, TAKENAKA K, MATSUMATA T, YOSHIDA Y, NISHIZAKI T, HASUO K AND SUGIMACHI K. (1989). A 5-year experience of lipiodolization. Selective regional chemotherapy for 200 patients with hepatocellular carcinoma. *Hepatology*, **10**, 98-102.

KAYE SB, CUMMINGS J AND KERR DJ. (1985). How much does liver disease affect the pharmacokinetics of adriamycin? *Eur. J. Cancer Clin. Oncol.*, **21**, 893-895.

KESTENS PJ, FARRELLY JA AND MCDERMOTT WV. (1961). A technique of isolation and perfusion of the canine liver. *J. Surg. Res.*, **1**, 58-63.

KOBAYASHI H, HIDAKA H, KAJIYA Y, TANOUE P, INOUE H, IKEDA K, NAKAJO M AND SHINOHARA S. (1986). Treatment of hepatocellular carcinoma by transarterial injection of anticancer agents in iodized oil suspension or of radioactive iodized oil solution. *Acta Radiol. Diagn.*, **27**, 139-147.

KU Y, SAITOH M, NISHIYAMA H, FUJIWARA S, IWASAKI T, TOMINAGA M, MAEKAWA Y, OHYANAGI H AND SAITOH Y. (1990). Extracorporeal removal of anticancer drugs in hepatic artery infusion. The effect of direct hemoperfusion combined with venovenous bypass. *Surgery*, **107**, 273-281.

LEGHA SS, BENJAMIN RS, MACKAY B, EWER M, WALLACE S, VALDIVIESO M, RASMUSSEN SL, BLUMENSCHEIN GR AND FREIREICH EJ. (1982). Reduction of doxorubicin cardiotoxicity by prolonged intravenous infusion. *Ann. Intern. Med.*, **96**, 133-139.

MARTINKOVA J, BULAS J, KREJCI V, HARTMAN M, TISLER I AND HULEK P. (1990). A study of the inhibition of adrenaline-induced vasoconstriction in the isolated perfused liver of rabbit. *Hepatology*, **12**, 1157-1165.

MOERTEL CG. (1987). An odyssey in the land of small tumors. *J. Clin. Oncol.*, **5**, 1502.

MUNCK J-N, RIGGI M, ROUGIER P, CHABOT GG, RAMIREZ LH, ZHAO Z, BOGNEL C, ARDOUIN P, HERAIT P AND GOUYETTE A. (1993). Pharmacokinetic and pharmacodynamic advantages of pirarubicin over Adriamycin after intraarterial hepatic administration in the rabbit VX2 tumor model. *Cancer Res.*, **53**, 1550-1554.

MYERS Jr CE AND CHABNER BA. (1990). Anthracyclines. In *Cancer Chemotherapy, Principles and Practice*, Chabner AC and Collins JM (eds). pp. 356-381. Lippincott: Philadelphia.

OKI T, KOMIYAMA T, TONE H AND INUI T. (1977). Reductive cleavage of anthracycline glycosides by microsomal NADPH cytochrome C reductase. *J. Antibiot.*, **30**, 613-615.

PELLETIER G, ROCHE A AND INK O. (1990). A randomized trial of hepatic arterial chemoembolization in patients with unresectable hepatocellular carcinoma. *J. Hepatol.*, **11**, 181-184.

PREISS R, MATTHIAS M, SOHR R, BROCKMAN B AND HULLER H. (1987). Pharmacokinetics of adriamycin, adriamycinol, and antipyrine in patients with moderate tumor involvement of the liver. *J. Cancer Res. Clin. Oncol.*, **113**, 593-598.

RUSZNIEWSKI P, ROUGIER P, ROCHE A, LEGMANN P, SIBERT A, HOCHLAF S, YCHOU M AND MIGNON M. (1993). Hepatic arterial chemoembolization in patients with liver metastases of endocrine tumors: a prospective phase II study in 24 patients. *Cancer*, **71**, 2624-2630.

SCHWEMMLE K AND AIGNER K. (1986). Requirements and results of liver perfusion. *Recent Results Cancer Res.*, **100**, 229-233.

SINDELAR WG. (1985). Isolation-perfusion of the liver with 5-fluorouracil. *Ann. Surg.*, **201**, 337-341.

SKIBBA JL, ALMAGRO UA, CONDON RE AND PETROFF RJ. (1983). A technique for isolation perfusion of the canine liver with survival. *J. Surg. Res.*, **34**, 123-132.

SKIBBA JL AND CONDON RE. (1993). Hyperthermic isolation-perfusion *in-vivo* of the canine liver. *Cancer*, **51**, 1303-1308.

TAKANASHI S AND BACHUR NR. (1976). Adriamycin metabolism in man. Evidence from urinary metabolites. *Drug Metab. Dispos.*, **4**, 79-87.

TERASAKI T, IGA T, SUGIYAMA Y AND HANANO M. (1989). Experimental evidence of characteristic tissue distribution of adriamycin, tissue DNA concentration as a determinant. *J. Pharm. Pharmacol.*, **34**, 597-600.

VAN DE VELDE CJH, KOTHUIS BJL, BARENBURG HWM, JONGEJAN N, RUNIA RD, DE BRAUW LM AND ZWAVELING A. (1986). A successful technique of in vivo isolated liver perfusion in pigs. *J. Surg. Res.*, **41**, 593-599.

VENOOK A, STAGG R, FRYE J, GORDEN R AND RING E. (1991). Chemoembolization of patients with liver metastases from carcinoid and islet cell tumors (abstract). *Proc. Am. Soc. Clin. Oncol.*, **10**, 386.